# Crosstalk between cystine and glutathione is critical for the regulation of amino acid signaling pathways and ferroptosis

**DOI:** 10.1038/srep30033

**Published:** 2016-07-18

**Authors:** Xinlei Yu, Yun Chau Long

**Affiliations:** 1 Department of Biochemistry, Yong Loo Lin School of Medicine, National University of Singapore, Singapore

## Abstract

Although essential amino acids regulate mechanistic target of rapamycin complex 1 (mTORC1) and the integrated stress response (ISR), the role of cysteine is unknown. We found that in hepatoma HepG2 cells, cystine (oxidized form of cysteine) activated mTORC1 and suppressed the ISR. Cystine deprivation induced GSH efflux and extracellular degradation, which aimed to restore cellular cysteine. Inhibition of γ-glutamyl transpeptidase (GGT) impaired the ability of GSH or cell-permeable GSH to restore mTORC1 signaling and the ISR, suggesting that the capacity of GSH to release cysteine, but not GSH per se, regulated the signaling networks. Inhibition of protein translation restored both mTORC1 signaling and the ISR during cystine starvation, suggesting the bulk of cellular cysteine was committed to the biosynthetic process. Cellular cysteine and GSH displayed overlapping protective roles in the suppression of ferroptosis, further supporting their cooperation in the regulation of cell signaling. Thus, cellular cysteine and its derivative GSH cooperate to regulate mTORC1 pathway, the ISR and ferroptosis.

The ability of cells to sense and respond to nutrient availability is critical for cell survival. It is well-established that essential amino acids are required for the regulation of protein translation and growth. Although cysteine is not considered an essential amino acid, cysteine deficiency is associated with various diseases including metabolic disorders, immune dysfunction, and cancer^1^.

Cysteine is oxidized to cystine which is readily transported into mammalian cells as a naturally occurring analog of cysteine^2^. In the cells, cystine is reduced back to cysteine, which is an essential substrate for the synthesis of biomolecules such as proteins, glutathione (GSH) and Coenzyme A^3^. GSH is a primary cellular antioxidant composed of glutamate, cysteine and glycine. It maintains the thiol status of critical proteins and defends against reactive oxygen species (ROS) via its reducing capacity^4^. GSH also exerts its cytoprotective function through conjugation reactions which mediates detoxification of xenobiotics and their metabolites. Although the function of GSH as an antioxidant or conjugate in detoxification has been extensively characterized, its role in the regulation of cystine-mediated signaling and cell growth is largely unknown. Mammalian cells are unable to produce cysteine *de novo* and the trans-sulfuration pathway which is required for the synthesis of cysteine from methionine is only present in the liver and a few other tissues^5^. Given that mammalian cells do not store a large level of cysteine, GSH may play a critical role in determining the cellular stress response during cysteine deficiency. The cysteine moiety of GSH can be liberated via γ-glutamyl cycle in which exported GSH is cleaved sequentially by two exofacial enzymes, namely γ-glutamyl transpeptidase (GGT) and dipeptidase (DP) to release cysteine which is then imported into the cells^6^. The first and rate-limiting step of GSH synthesis is catalyzed by glutamate-cysteine ligase, which is regulated by cysteine availability at the level of transcription and translation^6^. Although cysteine availability and GSH metabolism are tightly integrated, their cooperation in the regulation of amino acid sensing pathways and cell death is largely unknown.

The mechanistic target of rapamycin complex 1 (mTORC1) is a kinase which regulates anabolic metabolism, cell growth and proliferation^7^,^8^. Four canonical factors that are sensed by mTORC1 include amino acids, growth factors, energy status and oxygen level. Leucine, tryptophan, phenylalanine and arginine are identified as the best stimuli for mTORC1 activation^9^,^10^,^11^,^12^. The direct downstream targets of mTORC1 are ribosomal S6 kinase (p70S6K) and eukaryotic initiation factor 4E–binding proteins (4EBP) which regulate protein translation, cell size and cell cycle progression^7^,^8^. The ability of mTORC1 to sense the presence of amino acids and regulate protein translation ensures that cell metabolism is intimately coordinated with the macronutrient. Thus far, there is no report regarding the effect of cysteine or cystine on mTORC1 signaling.

Another network that integrates amino acid availability with cell physiology is the integrated stress response (ISR)^13^,^14^,^15^. Specific kinases are activated in response to different cellular stress in this system. For example, general control nonderepressible 2 (GCN2) is activated by amino acid starvation, whereas protein kinase-like endoplasmic reticulum kinase (PERK) is activated by endoplasmic reticulum (ER) stress^15^. The stress kinases in turn phosphorylate eukaryotic initiation factor 2α (eIF2α), and the collective effects of eIF2α activation is termed the ISR^14^. Phosphorylation of eIF2α leads to inhibition of general protein synthesis, but paradoxically increases the translation of selective mRNA such as ATF4^16^. The transcription factor ATF4 in turn activates stress-responsive genes such as Tribbles homolog 3 (Trib3) and solute carrier family 7 member 11 (Slc7A11) to mediate protective effects of the integrative stress response^16^. Although cysteine deprivation activates the ISR^14^, contribution of GSH to the regulation of the stress response remains largely unknown.

Deficiency of cellular cysteine induces a unique cell death program known as ferroptosis, a peroxidation-driven and iron-catalyzed form of non-apoptotic cell death^17^. It has been proposed that deficiency of intracellular cysteine induces ferroptosis primarily due to failure to synthesize GSH, which protects cells against ROS and lipid peroxidation^18^. Consistently, inhibition of cellular cysteine uptake by using erastin (an inhibitor of system x_c_^−^) depleted GSH and induced ferroptosis^19^. Depletion of cellular GSH by using buthionine sulfoxamine (BSO), which inhibits glutamate–cysteine ligase (the rate-limiting enzyme for GSH synthesis) is sufficient to induce ferroptosis due to impaired cellular antioxidant capacity^18^. Given that ROS that drive ferroptosis is reduced by GSH, the effect of cystine limitation on ferroptosis is mainly ascribed to the loss of antioxidant capacity. It is not known whether cysteine plays a primary role in the regulation of ferroptosis, beyond serving as a substrate in GSH synthesis.

Deficiency of cysteine and GSH are associated with numerous etiologically unrelated diseases including skeletal muscle wasting and cancer^1^. The “low CG syndrome” has been used to describe the symptoms associated with low cysteine and GSH, suggesting an overlapping role of the biomolecules in cellular function. In this study, we sought to investigate the role of cystine in the regulation of mTORC1, the ISR and ferroptosis, and whether GSH is an important determinant in the regulation independent of its reducing capacity.

## Results

### Cystine availability regulates mTORC1 signaling and the ISR

**Figure 1:**
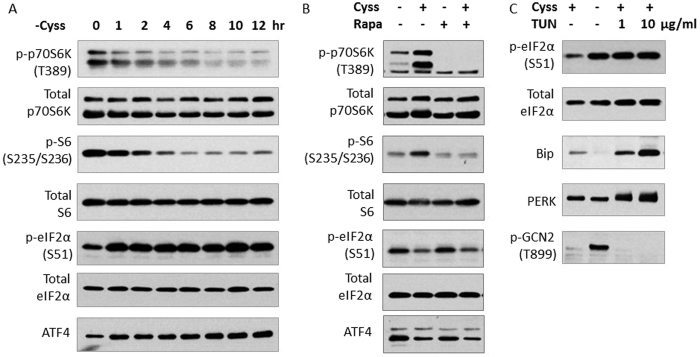
Cystine availability regulates mTORC1 signaling and the ISR. (**A**) HepG2 cells were cystine-starved for various durations. (**B**) HepG2 cells were deprived of cystine for 6 h before stimulated with or without cystine (200 μM) for 3 h. Rapamycin (20 nM) was added 1 hour prior to cystine (200 μM) stimulation. (**C**) Cells were treated with or without TUN (1 μg/ml or 10 μg/ml) in the presence or absence of cystine (100 μM) for 9 h. Immunoblotting was performed to evaluate the levels of phosphorylated and total proteins of mTORC1 pathway and the ISR.

We studied the effect of cystine starvation in HepG2 cells, which do not synthesize cysteine from methionine, to exclude the confounding effect of methionine^20^. Cystine deprivation resulted in a time-dependent reduction of mTORC1 signaling, as evidenced by the gradual decrease in the phosphorylation of p70S6K (T389) and S6 (S235/236), which are downstream effectors of mTORC1 (Fig. 1A). Cystine restriction elevated the level of phospho-eIF2α (S51) and ATF4 (Fig. 1A), major nodes of the ISR signaling. Thus, cystine limitation suppressed mTORC1 and activated the ISR, the two major amino acid sensing pathways in HepG2 cells.

We next asked the question whether the regulation of the ISR by cystine is mTORC1–dependent. We starved cells of cystine, and supplemented the media without or with cystine for 3 h. Supplementation of cystine restored mTORC1 signaling, and treatment with rapamycin (a specific mTORC1 inhibitor) abolished the induction, providing evidence for the direct role of mTORC1 in the phosphorylation of p70S6K (T389) and S6 (S235/236). Supplementation of cystine suppressed the ISR as evidenced by the reduction of phospho-eIF2α (S51) and ATF4, but rapamycin treatment had no discernible effect (Fig. 1B). The result suggested that cystine regulates the ISR independent of mTORC1 pathway in HepG2 cells.

Amino acid restriction and ER stress are two major stresses which activate the ISR and both pathways converge on eIF2α^14^. Given that cystine starvation is also known to induce ER stress^14^, we sought to evaluate which pathway is selectively activated by cystine starvation. GCN2 is a kinase which senses amino acid limitation, while PERK is activated in response to ER stress^15^. Cystine limitation increased phospho-GCN2 (T899), but not that of PERK (as no visible mobility shift was observed) (Fig. 1C). For comparison, we treated cells with tunicamycin (TUN, a widely-used ER stress inducer) which did not alter phospho-GCN2 (T899), but reduced the mobility of PERK, indicative of its increased phosphorylation (Fig. 1C). Bip (a downstream target of unfolded protein response, UPR) was elevated by tunicamycin but not cystine starvation (Fig. 1C). Thus, cystine starvation induces a stress signal of amino acid limitation through GCN2-eIF2α-ATF4 branch in the ISR but not ER stress in HepG2 cells.

### Cystine regulates cellular GSH homeostasis

**Figure 2:**
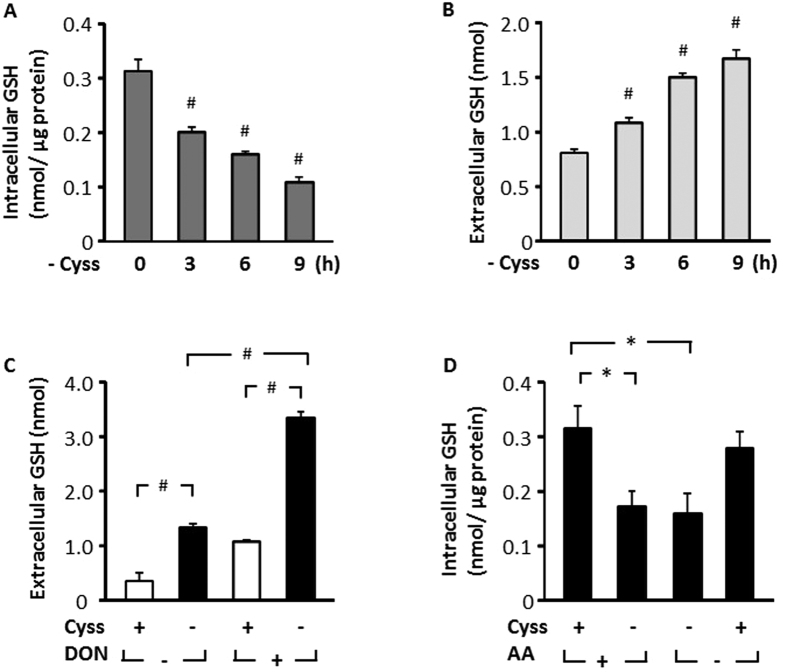
Cystine regulates cellular GSH homeostasis. (**A**,**B**) HepG2 cells were deprived of cystine in EBSS for various durations, and intracellular and extracellular GSH were assessed. (**C**) Cells were treated with or without cystine (100 μM) in the presence or absence of DON (1 mM) for 2 h and extracellular GSH was determined. (**D**) Cells were treated in EBSS with or without cystine (100 μM) in the presence or absence of 1 X MEM amino acids for 8 h, and intracellular GSH was evaluated. Data are expressed as means ± SEM of n = 3–6. *p < 0.05; ^#^p < 0.01.

Given that cysteine is an essential substrate for the synthesis of GSH^6^, we first evaluated whether intracellular GSH level is affected by cystine deprivation. Removal of cystine from culture medium led to a time-dependent decline of intracellular total GSH (inclusive of both GSH and GSSG, hereon referred to as GSH), with over 50% reduction at 9 hour (Fig. 2A). The decline in intracellular GSH coincided with a gradual increase of its accumulation in the medium (Fig. 2B), indicative of an elevation of GSH export in response to cystine starvation. The elevated export of GSH might reflect an adaptive response of the cell to supply cysteine via the γ-glutamyl cycle^6^. Consistently, when cells were subjected to cystine starvation in the presence of 6-diazo-5-oxo-L-norleucine (DON, a selective GGT inhibitor), there was a markedly higher accumulation of extracellular GSH (Fig. 2C). Thus, exported GSH was actively degraded by GGT. In order to determine whether cysteine is the primary amino acid which regulates intracellular GSH level, we starved cells of cystine in the presence or absence of other amino acids. Cystine starvation alone reduced GSH and was not further aggravated by deprivation of other amino acids, and supplementation of cystine alone was sufficient to restore GSH level in the absence of other amino acids (Fig. 2D). Thus, cystine is the primary amino acid which regulates cellular GSH homeostasis in HepG2 cells.

### GSH regulates mTORC1 signaling and the ISR during cystine starvation

**Figure 3:**
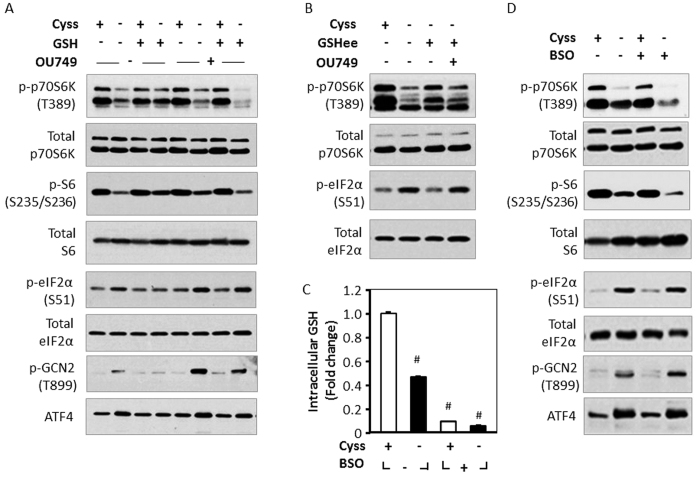
GSH regulates mTORC1 signaling and the ISR during cystine starvation. (**A**) HepG2 cells were treated with or without cystine (100 μM) in the presence or absence of OU749 (250 μm) for 9 h, and GSH (1 mM) was supplemented during the final 3 h for the indicated groups. (**B**) Cells were treated with or without cystine (100 μM) in the presence or absence of OU749 (250 μM) for 9 h, and GSHee (5 mM) was supplemented during the final 3 h for the indicated groups. Immunoblotting was performed to analyze the phosphorylated and total proteins. (**C**) Cells were pretreated with or without BSO (300 μM) for 18 h, and then stimulated with or without cystine (100 μM) in the presence or absence of BSO for (300 μM) 9 h. Intracellular GSH was measured with GSH colorimetric detection kit. Data are expressed as means ± SEM of n = 5, ^#^p < 0.01. (**D**) Cells were pretreated with or without BSO (300 μM) for 18 h, and then stimulated with or without cystine (100 μM) in the presence or absence of BSO (300 μM) for 4 h. Immunoblotting was performed to analyze the phosphorylated and total proteins.

Given that cystine and GSH balance are tightly coupled, we next evaluated whether GSH is a modulator of cystine-induced cell signaling. Under cystine-starved conditions, GSH supplementation rescued the phosphorylation of p70S6K (T389) and S6 (S235/236) of the mTORC1 pathway, and relieved the induction of phospho-GCN2 (T899), phospho-eIF2α (S51) and ATF4 of the ISR system (Fig. 3A). Inhibition of GSH degradation with OU749, a selective GGT inhibitor^21^, abolished the restorative effects of GSH on mTORC1 signaling and the ISR during cystine starvation (Fig. 3A). Thus, GSH degradation is required to prevent the stress response induced by cystine starvation in HepG2 cells.

We next asked whether the restorative effects of GSH on mTORC1 signaling and the ISR are mediated by GSH per se. To this end, we treated cystine-starved cells with GSHee, (a membrane-permeable analog of GSH), and found that GSHee rescued mTORC1 signaling and ISR (Fig. 3B). Consistent with our observation of GSH treatment, the preventive effects of GSHee were abrogated by OU749 (Fig. 3B). Therefore, GSH degradation (but not GSH per se) contributes to cysteine pool which regulates mTORC1 pathway and the ISR in HepG2 cells.

To determine the role of endogenous GSH under cystine deprivation, we treated cells with BSO (an inhibitor of GSH synthesis) in the presence or absence of cystine starvation. Treatment with BSO markedly reduced intracellular GSH level to less than 10% of control (Fig. 3C), independent of cystine availability. GSH depletion alone had no observable effect on mTORC1 signaling; however, it aggravated the decline in mTORC1 signaling under cystine limitation (Fig. 3D). Reduction of intracellular GSH alone did not activate the ISR, and cystine starvation induced a robust ISR which was not aggravated by BSO treatment (Fig. 3D). Thus, the ISR is more sensitive to cysteine insufficiency in comparison to GSH depletion. Taken together, our data provided evidence that GSH is a critical factor in cystine-regulated cell signaling, but GSH per se is not a direct regulator of the two pathways in HepG2 cells.

### GSH moderates the ISR gene expression during cystine starvation

**Figure 4:**
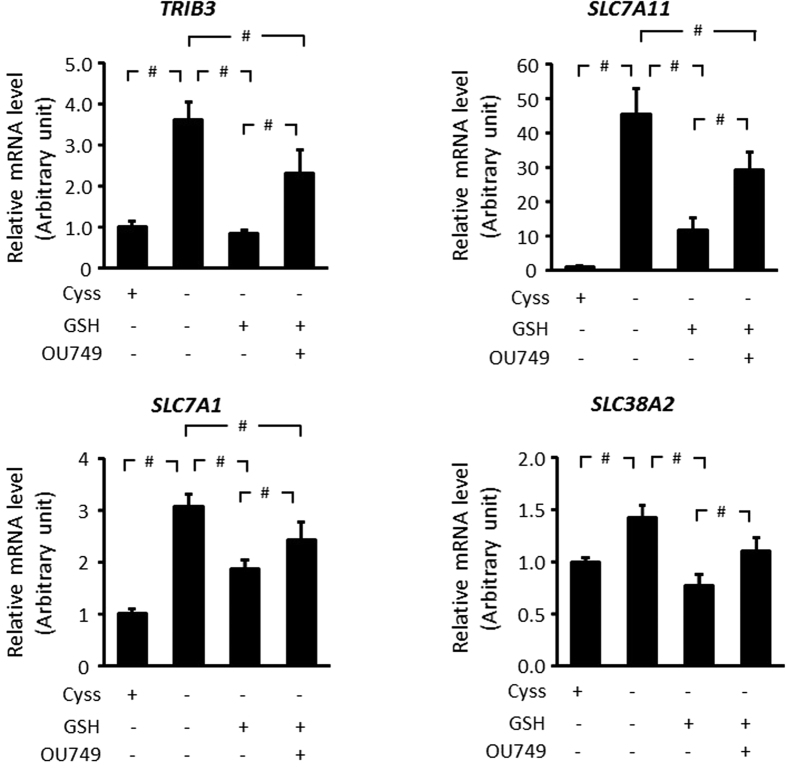
GSH moderates the ISR gene expression during cystine starvation. HepG2 cells were treated with or without cystine (100 μM) in the presence or absence of OU749 (250 μM) for 9 h and GSH (1 mM) was supplemented during the final 3 h. Relative mRNA levels of *TRIB3*, *SLC7A11*, *SLC7A1*, and *SLC38A2* were evaluated by real-time PCR and normalized against β-actin. Data are expressed as fold change compared to control. Data are the means ± SD for n = 6, ^#^p < 0.01.

Activation of the ISR elevates ATF4 to increase expression of genes which contain the amino acid response elements (AARE)^16^. Given that cystine starvation activated the GCN2-eIF2α-ATF4 axis, we evaluated the expression of several AARE-containing genes. The mRNA of *TRIB3*, *SLC7A11*, *SLC7A1*, and *SLC38A2* (which contain AARE in their promoter regions)^16^, was upregulated in response to cystine starvation. GSH supplementation prevented their induction in response to cystine starvation, but this effect was abrogated by OU749 (Fig. 4). The expression profile of these genes (Fig. 4) is consistent with the ISR signaling profile (Fig. 3A). Thus, cystine and GSH regulate the transcriptional program of the ISR in an interdependent manner in HepG2 cells.

### Inhibition of protein synthesis rescues both mTORC1 signaling and the ISR during cystine starvation

**Figure 5:**
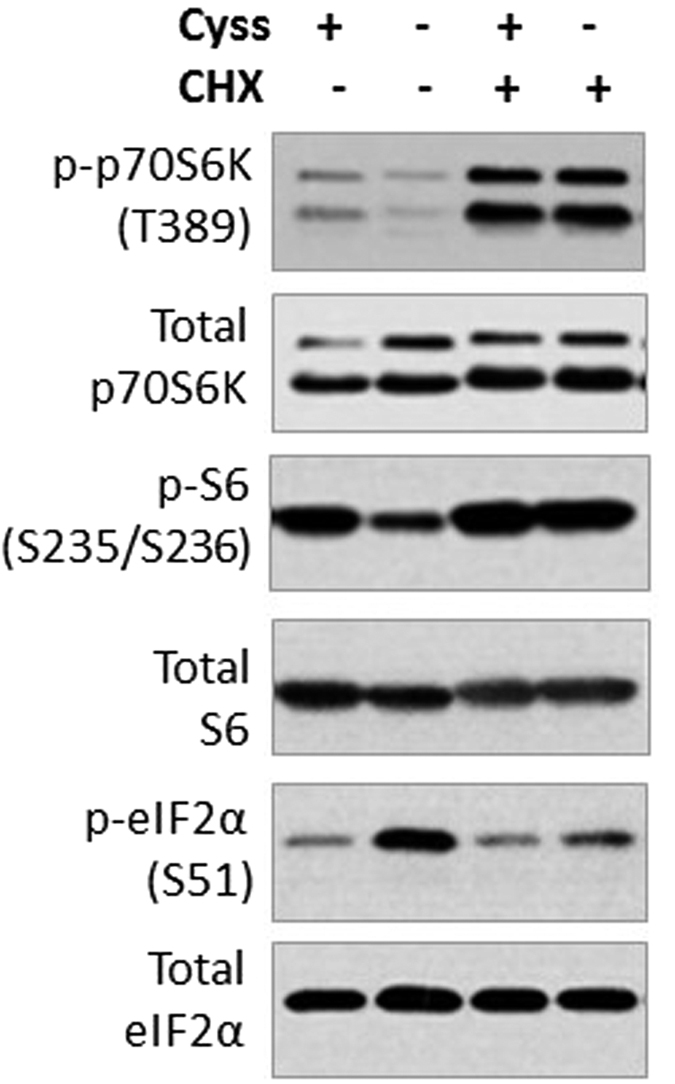
Inhibition of protein synthesis rescues both mTORC1 signaling and the ISR during cystine starvation. HepG2 cells were pretreated with or without CHX (2.5 μM), and then stimulated with or without cystine (100 μM) in the presence or absence of CHX (2.5 μM) for 6 h. Immunoblotting was performed to evaluate the level of phosphorylated and total proteins.

Protein synthesis takes precedence over other cysteine-consuming pathways under normal and particularly under sulfur-deficient conditions^22^. Our data showed that although degradation of GSH contributed to cysteine pool (Fig. 3), it was insufficient to maintain mTORC1 pathway and suppress the ISR during prolonged cystine limitation (Fig. 1). We speculated that the bulk of cysteine, including the fraction contributed by GSH, is utilized by protein translation which depletes intracellular cysteine pool. To test this possibility, we utilized cycloheximide (CHX) to inhibit protein synthesis. CHX restored mTORC1 signaling and relieved the ISR under cystine starvation (Fig. 5), suggesting that majority of cysteine is utilized for protein synthesis. The marked enhancement of mTORC1 signaling by CHX was also observed in the control cells (Fig. 5), indicating that protein synthesis is the predominant amino acid-consuming process in the cell. Our results provided evidence that inhibition of protein translation alone is sufficient to rescue both mTORC1 signaling and the ISR, suggesting its major role in the regulation of amino acid sensing pathways in HepG2 cells.

### Endogenous GSH protects cells against ferroptosis during cystine starvation

**Figure 6:**
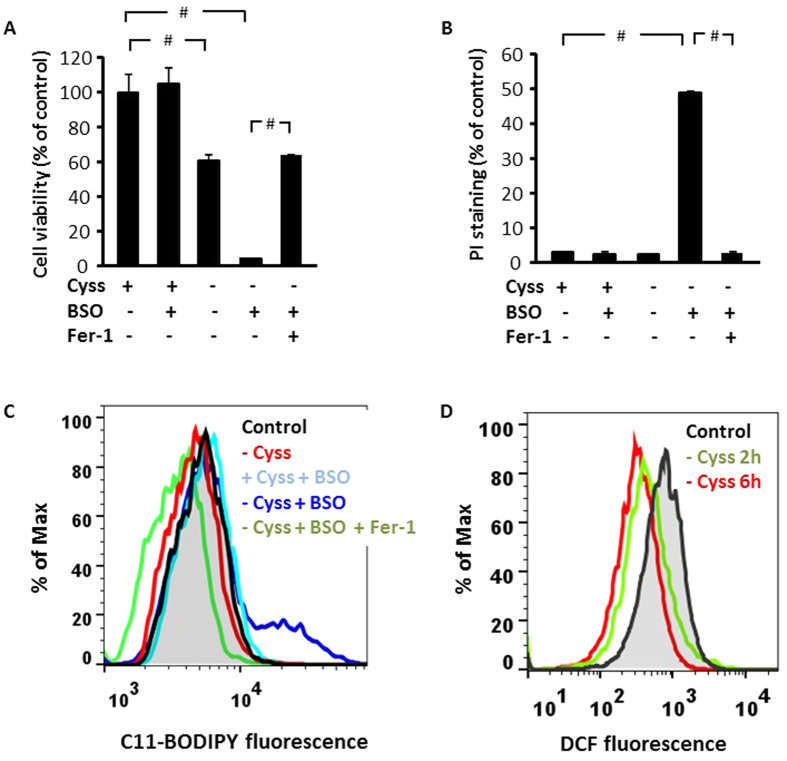
Endogenous GSH protects cells against ferroptosis during cystine starvation. (**A**,**B**) HepG2 cells were treated in DMEM (with 10% dialyzed FBS) with or without BSO (300 μM) in the presence or absence of cystine (100 μM) for 48 h, and Fer-1 (1μM) was added to the groups as indicated. Cell viability was evaluated by MTT assay, n = 6 (**A**), and the number of dead cells was assessed by using PI staining, n = 3 (**B**). Data are expressed as the means ± SEM, ^#^p < 0.01. (**C**) HepG2 cells were treated in phenol red-free DMEM (with 10% dialyzed FBS) with or without BSO (300 μM) in the presence or absence of cystine (100 μM) for 36 h. Fer-1 (1 μM) was added to the groups as indicated. The level of lipid ROS was assessed by flow cytometry using BODIPY 581/591 C11. (**D**) HepG2 cells were cystine starved for 2 or 6 h, and the level of soluble ROS was assessed by flow cytometry using CM-H2DCFDA. Representative result for 3 independent experiments is shown.

Our results thus far demonstrated the role of GSH in sustaining cystine-induced cell signaling when there is a shortage of the amino acid. We next examined the cooperation between GSH and cystine in the regulation of cell death. Depletion of intracellular GSH in HepG2 cells with BSO treatment was without effect on cell viability as assessed by MTT assay (Fig. 6A). Cystine starvation reduced cell viability which was severely aggravated by BSO treatment, supporting a protective role of GSH. Given that both cysteine and GSH contribute to cell metabolism, which may be a confounding factor in MTT assay, we verified the extent of cell death by propidium iodide (PI) staining. Cystine starvation and GSH depletion alone was without effect on the number of PI-positive cells (Fig. 6B). Consistent with our previous data which indicated a protective role of GSH under cystine-deprived conditions, cell death was most severe when both cystine and GSH were depleted in HepG2 cells (Fig. 6B).

Several inhibitors of cystine uptake are known to induce ferroptosis, an iron-dependent cell death program. Several features of ferroptosis include oxidative burst, particularly increased lipid peroxidation, and this cell death program can be readily prevented by a specific ferroptosis inhibitor, ferrostatin-1 (Fer-1)^19^. We found that cell death induced by cystine and GSH depletion was rescued by Fer-1 (Fig. 6A,B), suggesting a role of ferroptosis. We then evaluated the induction of lipid ROS in the cells to verify the activation of ferroptosis. We observed that lipid ROS were induced only in cells which were depleted of both cystine and GSH, and the induction was prevented by Fer-1 (Fig. 6C). Neither cystine starvation nor BSO-induced GSH depletion alone elevated lipid ROS (Fig. 6C). Although cystine starvation has been shown to induce oxidative stress, we did not observe any increase in soluble or lipid ROS production in cystine-starved cells (Fig. 6C,D). Thus, cystine and GSH cooperate to suppress lipid ROS and ferroptosis in HepG2 cells.

## Discussion

Cysteine is not categorized as an essential amino acid due to the presence of transsulfuration pathway in the liver^5^. However, hepatic production of cysteine is limited, and cysteine-deficient status is associated with numerous diseases such as insulin resistance, immune dysfunction, and cancer^1^. To gain insight into the role of cysteine in cellular physiology, characterization of cysteine-regulated cellular signaling is warranted.

The mTORC1 pathway and ISR are two major amino acid sensing pathways that integrate nutrient availability to cellular adaptations including protein synthesis, metabolism and cell growth^7^,^8^,^15^,^16^. Our study showed that HepG2 cells, which primarily rely on extracellular cystine as a source of cysteine^20^, responded to cystine deprivation by reducing anabolic signaling including attenuation of mTORC1 pathway and activation of the ISR (Fig. 1A). The inhibition of mTORC1 signaling by cystine starvation demonstrated that regulation of this pathway is not limited to essential amino acids^9^,^10^,^11^,^12^, but is also regulated by cysteine availability (Fig. 1A). mTORC1 phosphorylates and activates p70S6K which in turn phosphorylates ribosomal protein S6 to enhance translation efficiency^23^. Induction of protein translation will be a futile process when one of the 20 common proteogenic amino acids is deficient. Thus, the regulation of mTORC1 by cysteine availability is critical for cells to reduce global translation to conserve amino acids and energy. Consistent with the suppression of mTORC1 signaling, we observed that cystine starvation markedly activated the ISR (Fig. 1A), which inhibits global translation but selectively induces synthesis of numerous proteins involved in amino acid uptake^16^. Taken together, cystine starvation induced a signaling program that aims to restore cellular amino acid balance. Re-addition of cystine restored of mTORC1 signaling, which was abolished by rapamycin (Fig. 1B). Inhibition of mTORC1 pathway by rapamycin was without effect on the ISR, suggesting that mTORC1 signaling does not contribute to the ISR in this setting. This is in contrast with a previous report that showed mTORC1 inhibition activated the ISR^24^. The discrepancy could be due to the difference in cell models, and the high rapamycin concentration (100 nM) used in that study which may have induced cell stress^24^. Cystine starvation induced the phosphorylation of GCN2 but not PERK (Fig. 1C), suggesting that cystine starvation induced the amino acid response but not the ER stress signaling within the ISR. This is further supported by the lack of Bip upregulation, a hallmark feature of ER stress (Fig. 1C). Although GSH is involved in disulfide formation and protein folding in the ER^25^, our data showed that cystine starvation did not disrupt ER homeostasis in HepG2 cells.

Cysteine is required for the synthesis of GSH, an important antioxidant which regulates redox sensitive pathways including mTORC1 signaling and ISR^26^,^27^. In order to investigate the participation of GSH in cystine-induced cell signaling, we investigated the effects of cystine starvation on GSH homeostasis. Removal of extracellular cystine led to depletion of intracellular GSH and a corresponding increase in extracellular GSH accumulation (Fig. 2A,B). The data suggested that cells elevated GSH export in response to cystine limitation (Fig. 2B), to increase degradation of GSH via the γ-glutamyl cycle in an attempt to replenish cysteine. Consistent with the notion that GSH export and degradation are elevated during cystine starvation, there was a greater accumulation of extracellular GSH when GGT was inhibited (Fig. 2C), suggesting that a large proportion of exported GSH undergoes GGT-dependent degradation. Cystine appeared to be the main regulator of GSH homeostasis (among other amino acids), since cystine deprivation alone reduced intracellular GSH, and it was not further aggravated by depletion of other amino acids. Consistently, cystine supplementation alone was sufficient to sustain intracellular GSH in the absence of other amino acids (Fig. 2D). The results provided evidence that cystine is a major regulator of cellular GSH metabolism which may contribute to cystine-induced cell signaling in HepG2 cells.

Given that GSH is a critical cellular antioxidant, most of the regulatory effects of GSH on cell signaling are ascribed to its role in redox homeostasis^28^. Although GSH supplementation restored mTORC1 signaling and the ISR during cystine starvation, inhibition of GGT, the key enzyme responsible for ecto-degradation of GSH, abolished the rescue effects of GSH (Fig. 3A). Hence, the regulation of mTORC1 and the ISR by GSH requires its degradation, which releases and replenishes cysteine. The ability of GSH in reducing cystine starvation-induced phospho-GCN2, which detects uncharged-tRNA as a surrogate of amino acid deficiency^16^, provided further evidence that GSH participates in cell signaling as a reservoir of cysteine (Fig. 3A). To verify that GSH per se is not critical for mTORC1 and the ISR signaling, we incubated cells with GSHee, a cell-permeable GSH. Although GSHee was able to restore signaling of the two pathways, the effects were dependent on its degradation by GGT (Fig. 3B). Consistent with the notion that GSH per se does not regulate mTORC1 and the ISR, depletion of intracellular GSH exacerbated the loss of mTORC1 signaling upon cystine deprivation, but was without observable effect in the presence of cystine (Fig. 3C,D). Thus, GSH participates in cysteine-induced signaling via its degradation and ability to replenish cysteine pool in HepG2 cells. Cancer cells are known to contain high levels of GSH which supports cell proliferation, metastasis, and stress resistance^29^. It is also reported that numerous cancers are associated with overexpression of GGT, which participate in the recycling of cysteine from GSH^30^. Therefore, the role of GSH as a supply of cysteine may be as important, if not more crucial, to its role in redox homeostasis.

Cystine limitation induced the expression of AARE-containing genes in the ISR, which are involved in amino acid metabolism (*SLC7A11*, *SLC7A1*, and *SLC38A2*) and cell survival (*TRIB3*) (Fig. 4). The transcriptional program induced by cystine deprivation is similar to that of leucine starvation^14^, suggesting that the transcriptional program is a common adaptation of amino acid starvation to activate amino acid metabolism and stress resistance. Supplementation of GSH was sufficient to restore the gene expression level close to that of the control cells, while inhibition of GGT severely impaired the rescue (Fig. 4). The data suggested that release of the cysteine moiety of GSH is critical for the restoration of transcriptional response to cystine starvation, which corroborated the signaling result of p-GCN, p-eIF2α and notably ATF4, which induces the expression of genes containing AARE (Fig. 3A). Thus, GSH plays a critical role in the restoration of cell signaling and transcriptional program during cystine starvation in HepG2 cells.

Under sulfur-limiting conditions, cells utilize cysteine preferentially for protein synthesis to support fundamental biological processes^22^. This is supported at least in part at the biochemical level: the Km of L-cysteinyl-tRNA synthetase towards cysteine, which directs cysteine to translational machinery, is less than one tenth of that for other enzymes of cysteine metabolism such as GCS or cysteine dioxygenase^22^. Our data demonstrated that a great bulk of intracellular cysteine is used for protein synthesis during cysteine starvation in HepG2 cells (Fig. 5). When protein synthesis was blocked by CHX, sparing of cysteine from protein translation was sufficient to reactivate mTORC1 and relieve the ISR (Fig. 5). The large demand for cysteine by protein translation is a possible explanation for the weak restoration of cell signaling by endogenous GSH during cystine starvation (Fig. 1). Thus, there is considerable protein synthesis during cystine starvation which depletes amino acids and causes a reduction in mTORC1 signaling. One possible explanation for such a response is that inhibition of mTORC1 and activation of GCN2 collectively suppresses global translation to conserve nutrients, while selective translation of mRNA such as ATF4 (mediated by increased p-eIF2α) activates the expression of AARE genes to mitigate cellular stress.

HepG2 cells were able to survive prolonged cystine starvation or GSH depletion alone; however, there was severe cell death when both cystine and GSH were depleted (Fig. 6A,B). Given that pharmacological inhibition of cystine uptake induced ferroptosis^19^, we explored whether ferroptosis is induced by cystine- and GSH-depletion. Under dual depletion of cystine and GSH, HepG2 cells underwent ferroptosis, characterized by a marked increase in lipid ROS and rescue effects of Fer-1 (Fig. 6A–C). It has been reported that cystine starvation alone was sufficient to induce ferroptosis in mouse embryonic fibroblasts, which was associated with an outburst of ROS^31^. However, cystine starvation alone did not increase either soluble or lipid ROS in HepG2 cells (Fig. 6C,D), which might be due to the compensatory effects of GSH. The absence of oxidative stress presumably prevented cell death under cystine or GSH depletion alone, suggesting that cysteine and GSH cooperate to mitigate oxidative stress. Thus, the ability of cysteine and GSH to compensate one another to prevent ferroptosis is an important consideration in targeting the pathway for cancer therapy.

Our study demonstrated that cysteine (cystine) is an indispensable amino acid for mTORC1 signaling and the ISR in HepG2 cells. GSH preserves mTORC1 signaling activity and prevents the ISR during cystine deprivation, and this protective function is supported by its role as a cysteine storage molecule. Cystine starvation alone is not sufficient to induce ferroptosis because cysteine and GSH have compensatory roles in the prevention of ferroptosis. Therefore, inhibition of mTORC1 pathway and the induction of ISR may conceivably contribute to the pathological conditions related to cysteine deficiency.

## Materials and Methods

### Materials

Rapamycin (Rapa) was purchased from Tocris Bioscience (Bristol, UK). Cycloheximide (CHX), tunicamycin (TUN), 6-diazo-5-oxo-L-norleucine (DON), reduced L-glutathione (GSH), reduced glutathione ethyl ester (GSHee) were from Sigma-Aldrich (St. Louis, MO, USA). OU749, buthionine sulfoximine (BSO), and ferrostatin-1 (Fer-1) were from Cayman Chemicals (Ann Arbor, MI, USA). Antibodies specific for p70S6 kinase, phospho-p70S6 kinase (Thr 389), S6 ribosomal protein, phospho-S6 ribosomal protein (Ser 235/236), eIF2α, phospho-eIF2α (Ser 51), PERK, Bip and horseradish peroxidase (HRP)-linked secondary antibodies were from Cell Signaling Technology (Danvers, MA, USA). Antibody specific for phospho-GCN2 (Thr 899) was from Abcam (Cambridge, UK). ATF4 antibody was from Santa Cruz Biotechnology (Dallas, TX, USA).

### Treatment of cells

Hepatoma HepG2 cells were maintained in DMEM (GE Healthcare, Buckinghamshire, UK) supplemented with 10% fetal bovine serum (FBS), 100 U/ml penicillin, and 100 μg/ml streptomycin at 37 °C in 5% CO_2_. Unless indicated otherwise, cystine-free medium was prepared by supplementing cystine-, methionine- and glutamine-free DMEM (Gibco, CA, USA) with 100 μM methionine, 4 mM glutamine, 1 mM sodium pyruvate, 0.2% bovine serum albumin (BSA), 100 U/ml penicillin and 100 μg/ml streptomycin. The control medium contained 100 μM cystine. For cell viability assays, medium was supplemented with 10% dialyzed FBS. For GSH assay, cells were treated in Earle’s balanced salt solution (EBSS), supplemented with 25 mM glucose, 4 mM glutamine, 1 X MEM vitamin solution (Life technologies, CA, USA), 0.22% wt/vol NaHCO_3_, 1 mM sodium pyruvate, 0.2% BSA, 25 mM HEPES, 100 U/ml penicillin and 100 μg/ml streptomycin in the presence or absence of 1 X MEM amino acid solution (Gibco, CA, USA) with or without 100 μM cystine. For the measurement of ROS and lipid peroxidation, cells were treated in phenol red-free DMEM with 10% dialyzed FBS, 100 U/ml penicillin and 100 μg/ml streptomycin with or without cystine.

### Western Blot

HepG2 cells were homogenized on ice with RIPA buffer supplemented with phosphatase and protease inhibitors (Pierce, Thermo Scientific, MA, USA). Cell lysate was sonicated and centrifuged at 14,000 rpm at 4 °C to remove cell debris. Proteins were denatured in sample buffer and equal amounts were separated on SDS-PAGE and transferred to PVDF membrane (BioRad Laboratories, CA, USA). Membranes were blocked with 5% skimmed milk in Tris-buffered saline containing 0.1% Tween 20 for 1 hour at room temperature, and incubated with primary antibodies overnight at 4 °C. HRP-conjugated secondary antibody was used to detect the protein with chemiluminescence.

### Quantitative real-time PCR

RNA was purified by using ReliaPrep RNA (Promega, WI, USA) and reverse transcription was performed using 1.5 μg total RNA as template with the ImProm-II Reverse Transcription System (Promega, WI, USA). Real-time PCR was performed on ABI 7300 Real-Time PCR System (Life technologies, CA, USA) using SYBR Green Select Master Mix (Applied Biosystems, CA, USA). Data was analyzed using the relative quantitative method, by normalizing the mRNA level of all genes against that of β-actin gene.

### Measurement of GSH

Medium was collected for the determination of extracellular total GSH level, and cells were homogenized in 5% 5-sulfosalicylic acid dihydrate (SSA, Sigma-Aldrich, MO, USA) on ice. Supernatant was collected after centrifugation for the measurement of intracellular GSH. The pellet was dissolved in 100 μl RIPA buffer and protein content was determined. Total GSH was measured using Glutathione Colorimetric Detection Kit (Arbor Assays, Michigan, USA) according to the manufacturer’s instructions.

### Measurement of reactive oxygen species and lipid peroxidation

Cells were incubated with 5 μM BODIPY 581/591 C11 (Life technologies) for lipid peroxidation, or 5 μM CM-H2DCFDA (Life technologies) for ROS in the dark for 30 minutes at 37 °C. Cells were then harvested by trypsinization, washed once with phosphate-buffered saline (PBS), and re-suspended in 300 μl phenol red-free DMEM with 0.2% bovine serum albumin. Fluorescence was analyzed using a flow cytometer (BD FACSCanto™ II, BD Biosciences, Franklin Lakes, NJ, USA) equipped with a 488 nm laser for excitation. Data were collected using the 530/30 nm band-pass filter.

### Cell Viability Assays

Cell were treated in 96-well plate for 48 h, and incubated with 5 mg/ml 3-(4,5-dimethylthiazol-2-yl)-2,5-diphenyltetrazolium bromide (MTT, Sigma-Aldrich) for 4 h at 37 °C in the dark. After medium was removed, DMSO was added to each well to dissolve the formazan crystals, and absorbance at 540 nm was measured.

### Determination of cell death by PI staining

Cells were trypsinized, washed with PBS, and suspended in 300 μl Dulbecco’s phosphate buffered saline (DPBS) supplemented with 0.2% BSA containing 0.3 μg/ml propidium iodine (PI, Sigma-Aldrich). After 30 minutes of incubation at 37 °C in the dark, the percentage of PI positive cells was determined by using a flow cytometer (BD FACSCanto™ II, BD Biosciences), with 488 nm excitation laser and 585/42 nm band pass filter. The percentage of dead cells was presented as % of PI positive cells based on 10,000 events per sample.

### Statistical Analysis

Data are presented as mean ± SEM or ± SD for at least 3 independent replicates. Differences among groups were determined by one-way ANOVA followed by Fisher’s least significant differences *post hoc* analysis. Significance was accepted at p < 0.05.

## Additional Information

**How to cite this article**: Yu, X. and Long, Y. C. Crosstalk between cystine and glutathione is critical for the regulation of amino acid signaling pathways and ferroptosis. *Sci. Rep.* **6**, 30033; doi: 10.1038/srep30033 (2016).
